# Modulating redox homeostasis and cellular reprogramming through inhibited methylenetetrahydrofolate dehydrogenase 2 enzymatic activities in lung cancer

**DOI:** 10.18632/aging.103471

**Published:** 2020-08-06

**Authors:** Chun-Hao Chan, Chia-Yu Wu, Navneet Kumar Dubey, Hong-Jian Wei, Jui-Hua Lu, Samantha Mao, Joy Liang, Yu-Hsuan Liang, Hsin-Chung Cheng, Win-Ping Deng

**Affiliations:** 1 School of Dentistry, College of Oral Medicine, Taipei Medical University, Taipei 11031, Taiwan; 2 Stem Cell Research Center, College of Oral Medicine, Taipei Medical University, Taipei 11031, Taiwan; 3 Division of Oral and Maxillofacial Surgery, Department of Dentistry, Taipei Medical University Hospital, Taipei 11031, Taiwan; 4 School of Dental Technology, College of Oral Medicine, Taipei Medical University, Taipei 11031, Taiwan; 5 Department of Materials Science and Engineering, National Taiwan University, Taipei 10617, Taiwan; 6 Department of Dentistry, Taipei Medical University Hospital, Taipei 110131, Taiwan; 7 Graduate Institute of Basic Medicine, Fu Jen Catholic University, New Taipei 242, Taiwan

**Keywords:** MTHFD2, lung cancer, oxygen tension, tumorigenicity

## Abstract

Recent reports have indicated the role of highly expressed methylenetetrahydrofolate dehydrogenase 2 (MTHFD2) enzyme in cancers, showing poor survival; however, detailed mechanistic insight of metabolic functions of MTHFD2 have not been well-defined. Therefore, we aimed to examine the metabolic functions and cellular reprograming potential of MTHFD2 in lung cancer (LCa). In this study, we initially confirmed the expression levels of MTHFD2 in LCa not only in tissue and Oncomine^TM^ database, but also at molecular levels. Further, we reprogrammed metabolic activities in these cells through MTHFD2 gene knockdown via lentiviral transduction, and assessed their viability, transformation and self-renewal ability. In vivo tumorigenicity was also evaluated in NOD/SCID mice. Results showed that MTHFD2 was highly expressed in stage-dependent LCa tissues as well in cell lines, A549, H1299 and H441. Cellular viability, transformation and self-renewal abilities were significantly inhibited in MTHFD2-knockdown LCa cell lines. These cells also showed suppressed tumor-initiating ability and reduced tumor size compared to vector controls. Under low oxygen tension, MTHFD2-knockdown groups showed no significant increase in sphere formation, and hence the stemness. Conclusively, the suppressed levels of MTHFD2 is essential for cellular metabolic reprogramming leading to inhibited LCa growth and tumor aggressiveness.

## INTRODUCTION

Cancer is one of the known leading cause of most deaths all over the world. Specifically, according to National Cancer Institute report, there were 14 million new cases and 8.2 million cancer-related deaths worldwide in 2012 [1]. This may be ascribed to high possibility of cancer to metastasize, and to relapse after surgery, leading to failure in achieving cure following surgery, as evidenced in many cases to achieve a cure [2]. Current approaches to eliminate cancer include chemotherapy, radiotherapy and their combination, which might decrease the chance to relapse. However, no superior survival rate in patients after these therapies have been reported [3–5]. Therefore, a comprehensive treatment of cancer is urgently needed.

Cancer metastasis and relapse are now often attributed to cancer stemness, which impart self-renewal and unlimited differentiation capacity to cancer cells [6]. These cells in tumor tissues proliferate rapidly, and eventually the nutrient and oxygen supply from normal vasculature would be exhausted by the cells quickly, leading to low oxygen tension, during which hypoxia-inducible factors (HIFs) would stimulate various distinct pathways. HIF-1α is the master regulator and plays a fundamental role in hypoxic responses, and only factor involved in regulating glycolytic pathway [7]. It has been reported that up-regulated levels of HIF-1α, impacts the expression of many representative target genes, associated with cellular function, which terminally induce cellular apoptosis, metastasis, angiogenesis and inhibit mitochondrial respiration and differentiation [8]. In a seminal study by Keith et al., HIF-1α is might be considered as a potential therapy to eliminate cancer stemness in tumor [9, 10].

Besides, during rapid cancer cell proliferation, the replication of nucleic acid is an essential factor. The folate-pathway within one carbon cycle is the key pathway for producing nucleotides like purines and thymidylate, which are required for DNA synthesis. In folate cycle, metabolic gene methylenetetrahydrofolate dehydrogenase 2 (MTHFD2) plays an important role on purine synthesis. MTHFD2 is a bifunctional enzyme that is integral to mitochondrial one-carbon metabolism, particularly in folate cycle, MTHFD2 could catalyze NAD^+^ dependent 5,10-Methy-lenetetrahydrofolate (5,10-CH_2_-THF) dehydrogenase/ 5,10-methenyltetrahy-drofolate (5,10-CH^+^-THF) cyclohydrolase then form 10-formyltetrahydrofolate (10-CHO-THF) within mitochondria, which is important for purines synthesis in cytosol [11, 12]. In 2014, Roland Nilsson et al. compared mRNA profiles of 1454 metabolic enzymes across 1981 tumors spanning 19 cancer types. Among the top 50 metabolic enzymes, MTHFD2 frequently overexpressed the most in human tumors. Besides, metabolic reprogramming and redox homeostasis are closely associated and stimulate cancer progression by either activating proto-oncogenes or repressing onco-suppressors [13]. Studies have also indicated that folate-pathway contributes to majority of cellular production of NADPH [14, 15], which is a crucial element in anti-oxidation systems [16]. Moreover, previous studies have not only indicated a significant correlation between MTHFD2 expression and cancer proliferation [17], but also MTHFD2-mediated regulation of cell motility and invasion in breast cancer [18]. These above-mentioned evidences imply that MTHFD2 may act as a target to suppress cancer stemness, and hence the tumor aggressiveness. Therefore, we investigated the impact of MTHFD2 knockdown on cellular metabolic reprograming and redox homeostasis in LCa.

## RESULTS

### Expression profile of MTHFD2 in lung cancer (LCa)

**Figure 1:**
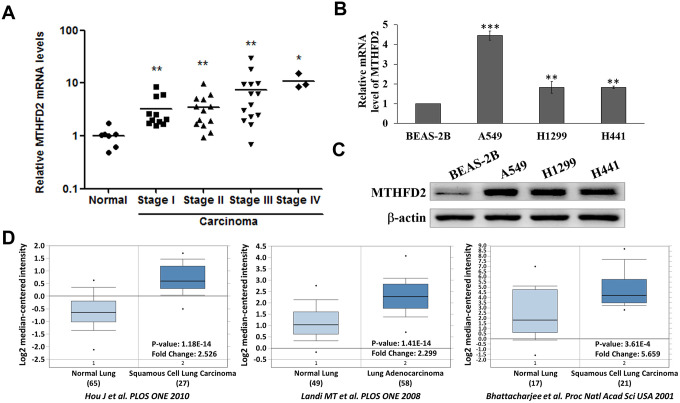
**Levels of MTHFD2 in lung cancer tissues and cell lines.** (**A**) Relative MTHFD2 gene expression of normal lung tissues and the different stages of lung cancer tissues. * and ** indicate *p*<0.05 and *p*<0.01, respectively using paired *t-test*, compared to normal group. (**B**) Relative MTHFD2 gene expression of normal lung cell line BEAS-2B and lung cancer cell lines A549, H1299 and H441. ** and *** indicate *p*<0.01 and *p*<0.001, respectively using one-way ANOVA, compared to normal cell line BEAS-2B. (**C**) Representative MTHFD2 protein expression in normal and lung cancer cell lines, where β-actin was loaded as relative control (**D**) Online Oncomine database-dependent (https://www.oncomine.org/) *in silico* analysis of fold changes in MTHFD2 expression in lung cancer tissues. MTHFD2 mRNA expression in normal and malignant lung specimens are presented as box and whisker plots. Sample numbers, fold changes, and *p-value* for MTHFD2 expression between normal and malignant specimens are indicated. Data are expressed as mean ± SD.

Cancers are presumed to proceed successively from less to more advanced preclinical phases. Additionally, their stage-specific correlation with metabolic gene MTHFD2 mediating mitochondrial folate pathway is still unknown. Our data showed a significantly increased expression profile of MTHFD2 mRNA in all stages of LCa tissue compared to normal with increasing tendency (Figure 1A). Similar results were evident in mRNA (Figure 1B) and protein (Figure 1C) levels in three human LCa cell lines A549, H1299 and H441 compared with normal lung epithelial cell line. These outcomes were further corroborated using Oncomine^TM^ Database in three previous studies [19–21], showing highly significant expression (over 2-fold) of MTHFD2 in LCa compared to normal counterparts (Figure 1D). A similar trend of MTHFD2 expression profiles was also observed in The Cancer Genome Atlas (TCGA) and genotype-tissue expression (GTEx) projects using GEPIA2 online platform (http://gepia2.cancer-pku.cn/#index) (Supplementary Figure 1), which further validates expression levels.

### Impact of MTHFD2 knockdown on LCa growth and proliferation

**Figure 2:**
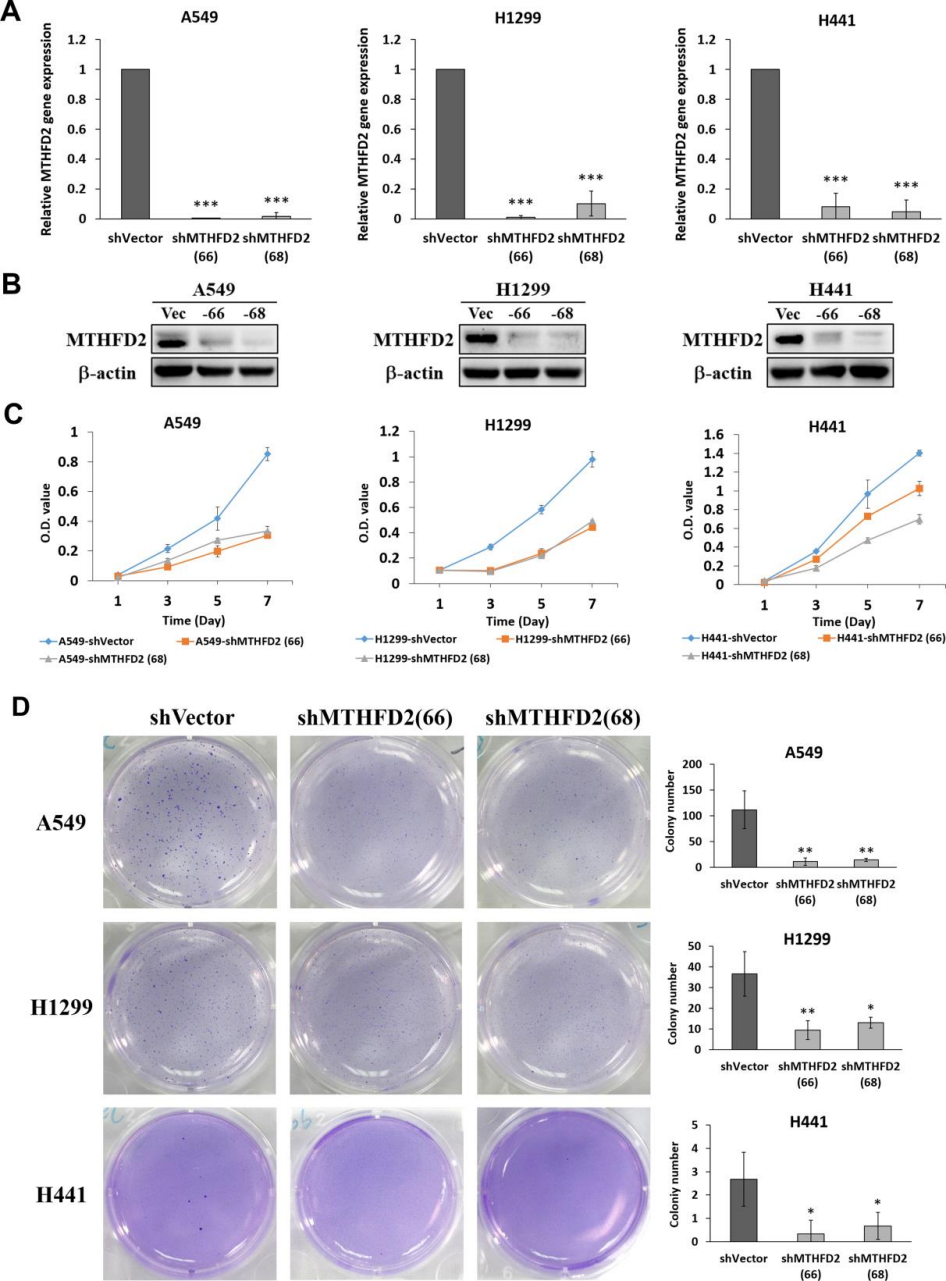
**MTHFD2 knockdown on growth and proliferation potential of lung cancer.** (**A**) Relative gene expression of MTHFD2 in knockdown group compared to vector control. *** indicate p<0.001 using one-way ANOVA. (**B**) The MTHFD2 protein expression of vector control group and MTHFD2-knockdown group, where β-actin as loading control. (**C**) MTT assay-dependent cell viabilities of MTHFD2-knockdown groups of A549, H1299 and H441 compared to vector control. (**D**) The morphology and quantification of formed colonies of vector control and MTHFD2-knockdown groups of A549, H1299 and H441 cell lines by soft agar assay. * and ** indicate p<0.05 and p<0.01, respectively using one-way ANOVA, compared to shVector group.

Mitochondrial folate-coupled metabolism is thought to be central in proliferative cancer [22]. Hence, we investigated the role of MTHFD2 in LCa growth and proliferation by establishing vector control and MTHFD2-knockdown cell lines of A549, H1299 and H441 through lentiviral transduction. The efficiency of these cells lines was examined by detecting GFP expression through flow cytometry (Supplementary Figure 2), and the influence of lentiviral transduction to the cell viability was evaluated through MTT assay (Supplementary Figure 3). Our results confirmed gene (Figure 2A) and protein (Figure 2B) levels of expressed MTHFD2, showing clearly established cell lines. Further, MTT assay demonstrated significantly inhibited proliferation ability of MTHFD2-knockdown cell lines (Figure 2C). Eventually, soft agar exhibited significantly decreased number of colonies in all the MTHFD2-knockdown cell lines (Figure 2D), indicating the role of MTHFD2 in malignant transformed cells. However, the cytometric analysis of apoptosis showed that compared to vector control, no significant increase in apoptotic population in early (Q4) as well as late (Q2) phase of MTHFD2-knockdown cell lines was observed (Supplementary Figure 4). This indicate that MTHFD2 knockdown may not directly cause significant cell apoptosis, but might decelerate cell growth ability, which also corresponds to our results of MTT assay and soft agar assay.

### MTHFD2-mediated lung cancer stemness

**Figure 3:**
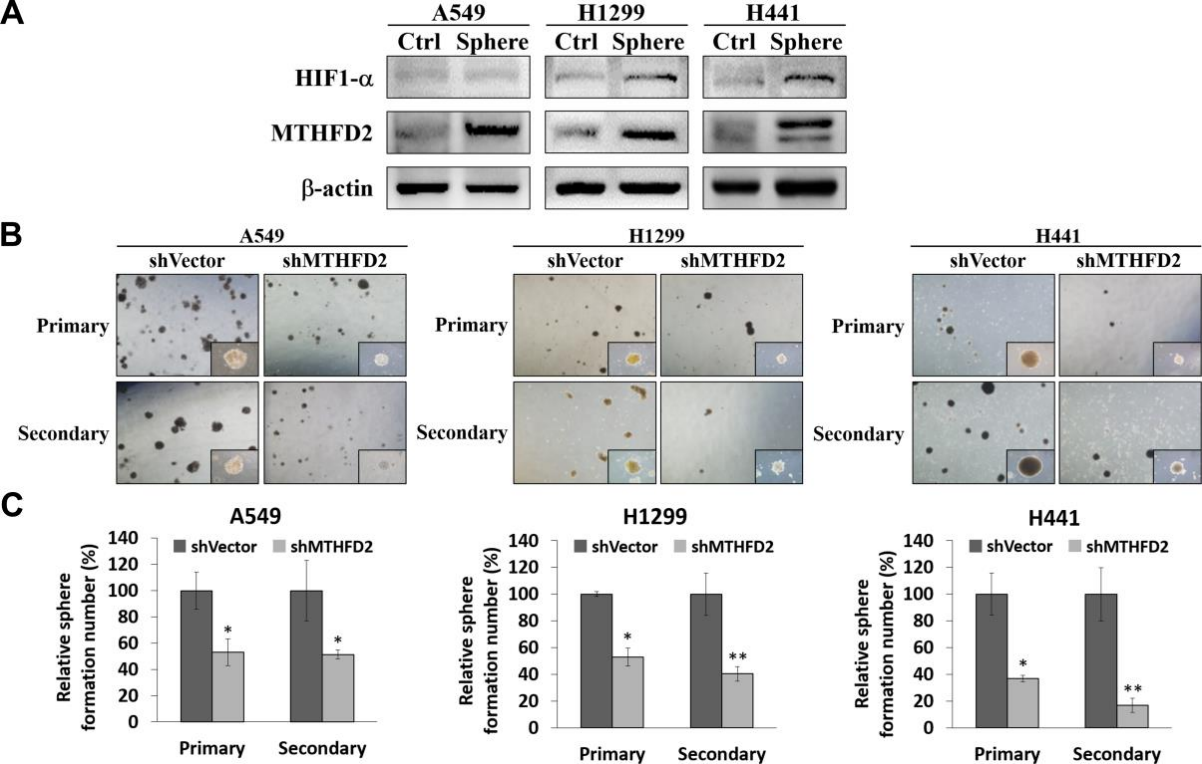
**MTHFD2 knockdown and assessment of lung cancer stemness through sphere formation assay.** (**A**) Representative protein expression of MTHFD2 and HIF-1α in adherent non-sphere forming cells (Ctrl) and sphere cells (Sphere) of parental cell lines, β-actin was used as loading control. (**B**) Bright-field images of primary and secondary tumor spheres (at 4X and 10X magnifications) and (**C**) their relative quantification of vector control and MTHFD2-knockdown cell lines, A549, H1299 and H441. * and ** indicate p<0.05 and p<0.01, respectively using paired *t-test*.

To deduce the relation between MTHFD2 and LCa cell stemness, we conducted sphere formation assay, which is widely used to identify stem cells based on their self-renewal and differentiation potential [23]. Our results showed significantly up-regulated protein expressions of both MTHFD2 as well as HIF-1α in parental A549, H1299 and H441 sphere-forming cells compared to counterparts (Figure 3A), implying that LCa stemness might be attributed to increased MTHFD2 expression, possibly through inducing elevated levels of HIF-1α under low oxygen tension.

To further investigate whether MTHFD2 modulate LCa self-renewal ability, vector control and MTHFD2-knockdown group were conducted with primary sphere formation to evaluate cancer stem cell population and secondary sphere formation for detecting maintenance of stemness respectively. We found that using equal concentration of cells seeded for formation of primary and secondary spheres, both the number as well as size of formed spheres were significantly inhibited in MTHFD2-knockdown cell lines (Figure 3B and 3C). This indicate that MTHFD2 is vital for self-renewal and differentiation ability of LCa cells.

### Impact of MTHFD2 on cellular metabolic reprogramming-dependent tumor aggressiveness

**Figure 4:**
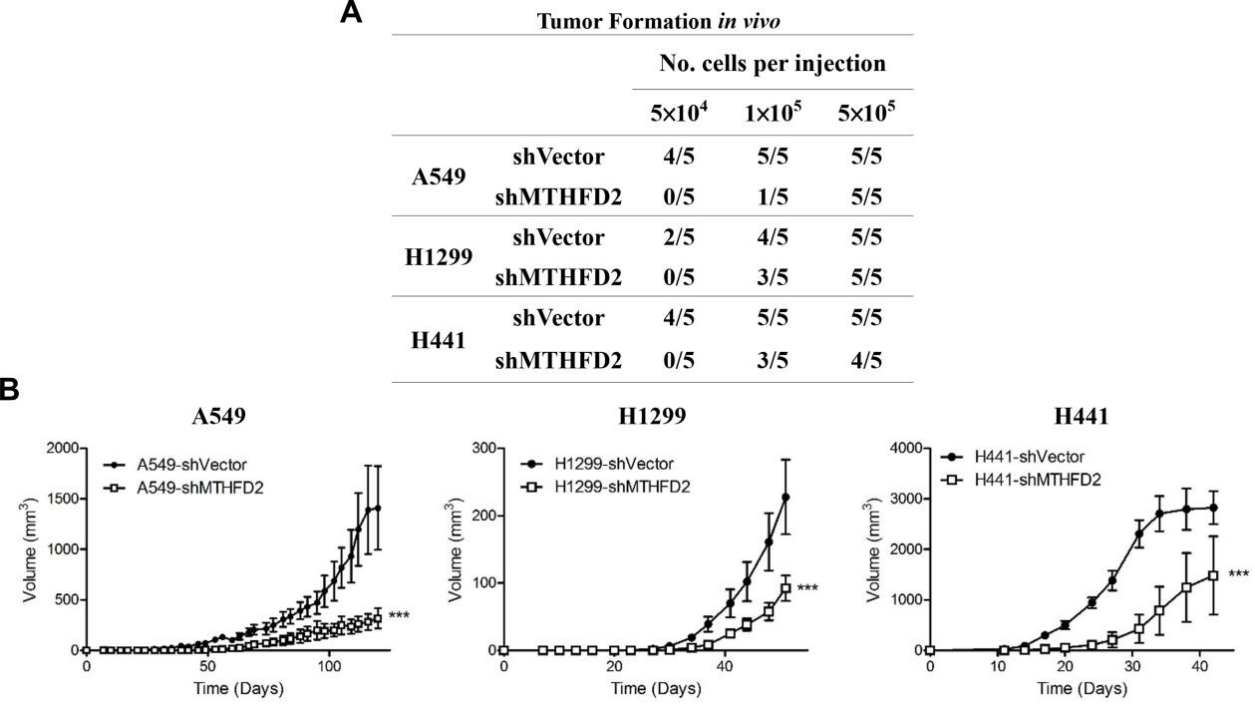
**MTHFD2 knockdown and *in vivo* tumor formation ability.** The cancer initiating ability (**A**), and tumor volumes (**B**) after subcutaneous injection of different cell number of respective vector control and MTHFD2-knockdown cell lines of A549, H1299 and H441 into the right flank of 6-week-old NOD/SCID mice (n = 5). *** indicate *p*<0.001 using one-way ANOVA, compared to vector control group.

Besides determining cancer stemness through *in vitro* sphere formation, an *in vivo* limiting dilution transplantation assay is highly important to assess the minimal amount of cells capable of initiating LCa, which was done by subcutaneous injection of various dilutions of vector control and MTHFD2-knockdown LCa cells lines in NOD/SCID mice. The results demonstrated that in 5×10^4^ inoculated cells group, more tumors were formed in vector controls (A549 (80%), H1299 (40%) and H441 (80%)), whereas no tumors were formed in MTHFD2-knockdown groups (Figure 4A). Besides, though injected cells at same concentration (5×10^5^) potentially formed tumors in all mice, the tumor size of vector control was significantly larger compared to MTHFD2-knockdown groups (Figure 4B). This indicate the possible role of MTHFD2 knockdown to metabolically reprogram the aggressive phenotypes of LCa cells to a lesser extent.

### Cellular oxygen sensing and MTHFD2-mediated metabolic reprogramming

**Figure 5:**
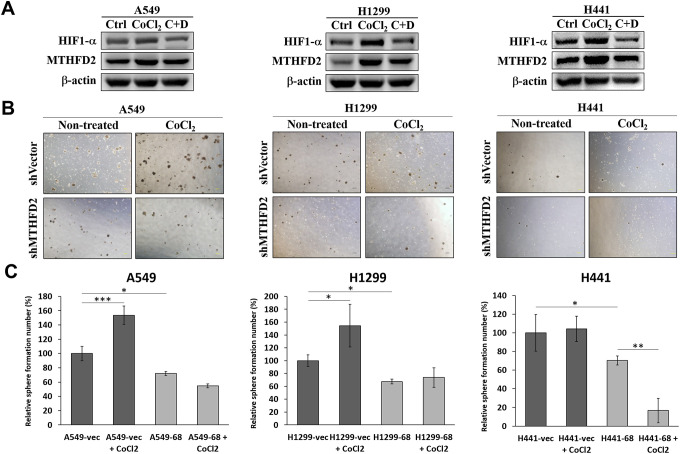
**Oxygen availability and regulation of MTHFD2.** (**A**) Representative western blots showing hypoxia inducible factor (HIF)-1α and MTHFD2 protein expression in A549, H1299 and H441 cells after treatment with only 100 μM CoCl2 or combined with 100nM digoxin (C+D), where β-actin was used as loading control. (**B**) Bright-field images of sphere formed by vector control and MTHFD2-knockdown groups of A549, H1299 and H441 cells and (**C**) their relative quantified number in the presence or absence of 100 μM CoCl2 for 2 days. *, ** and *** indicate p<0.05, p<0.01 and p<0.001, respectively using one-way ANOVA, compared to vector control group.

Available oxygen level and its sensing induce notable and clinically relevant alterations in cancerous cells and tumors by controlling gene expression [24]. Hence, we explored whether mitochondrial folate microenvironment is associated with cellular oxygen sensing and MTHFD2. We treated parental A549, H1299 and H441 cell lines with cobalt chloride (CoCl_2_) and digoxin, the low oxygen tension-mimetic agent and hypoxia-inducible factor (HIF)-1α inhibitor respectively, and their combination as well. Our data showed that in the presence of CoCl_2_, levels of HIF-1α and MTHFD2 were increased, which were suppressed in presence of digoxin (Figure 5A). Also, HIF-1α and MTHFD2 levels were time-dependently increased following CoCl_2_ treatment (Supplementary Figure 5). This infer that CoCl_2_ may decrease cellular oxygen sensing potential, leading to elevated levels of HIF-1α and MTHFD2. Further, sphere formation assay was conducted after the cell lines were pre-treated with CoCl_2_ for 48 hours. The results demonstrated that only vector control groups showed significantly increased sphere number, whereas MTHFD2-knockdown groups showed no significant increase compared to CoCl_2_-treated vector group (Figure 5B and 5C). This was further corroborated by examining protein levels of HIF-1α and MTHFD2 in vector control and MTHFD2-knockdown groups after CoCl_2_ treatment, which was significantly increased only in vector control groups, supporting highly formed spheres in this group (Supplementary Figure 6).

### Influence of MTHFD2 on oxidative stress response and metabolic reprogramming in lung cancer

**Figure 6:**
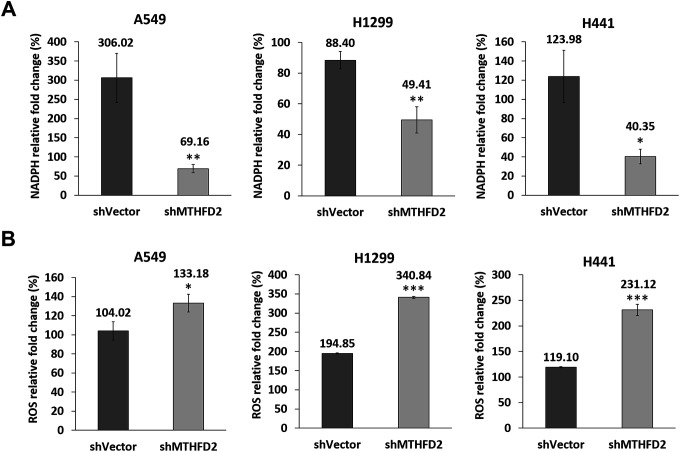
**MTHFD2 and maintenance of redox homeostasis under low oxygen tension.** Rate of change (%) of (**A**) NADPH and (**B**) ROS production in vector control and MTHFD2-knockdown groups of A549, H1299 and H441 cells after their treatment with 200 μM of CoCl_2_ for 24 hours. *, ** and *** indicate p<0.05, p<0.01 and p<0.001, respectively using paired *t-test*, compared to shVector group.

To investigate the MTHFD2-mediated redox homeostasis under low oxygen tension, we determined the levels of NADPH and ROS after treating vector control and MTHFD2-knockdown groups with CoCl_2_. The vector control groups revealed significantly higher expression of NADPH (Figure 6A); whereas ROS level was significantly increased in MTHFD2-knockdown groups (Figure 6B). These results imply that MTHFD2 might contribute in maintaining cellular redox homeostasis under low oxygen tension, knockdown of which led to accumulated ROS levels, resulting into inhibited LCa phenotype.

## DISCUSSION

The current study has identified the potential role of MTHFD2 in LCa through its expression patterns in tissues and cell lines. We also demonstrated how knockdown of MTHFD2 influence the LCa characteristics such as cell proliferation and viability, metabolic reprogramming, stemness, cellular oxygen sensing and response under oxidative stress. Genomic instability may commence cancer, aggravate progression, and impact the altogether prognosis of affected patients [25]. Initially, we correlated MTHFD2 genomic instability with stage-specific LCa tissues, which was significantly higher with increasing tendency in all stages than those with normal. This was further supported by increased mRNA levels of MTHFD2 in three human LCa cell lines A549, H1299 and H441 and Oncomine^TM^ Database in three previous studies [19–21]. These evidences identify MTHFD2 as a molecular signature for cancer patient stratification. Based on evident increase of MTHFD2 in LCa, we next attempted to understand the effect of loss of MTHFD2, and how it orchestrates the profound metabolic reprogramming required for LCa growth. Our results revealed not only diminished levels of MTHFD2 gene and protein but also the proliferation ability and the number of soft agar colonies of MTHFD2-knockdown cell lines, implying that MTHFD2 imparted malignancy to transformed cells. A supportive evidence showed that suppressed MTHFD2 levels reduced leukemia burden [26]. On contrary, study has also demonstrated contradictory findings in which MTHFD2-knockdown did not significantly alter cell proliferation or induction of apoptosis in breast cancer cells (MDA-MB-231) [18]; however, it regulated cell motility and invasion through disrupting vimentin network formation and reducing N-cadherin expression, which are the key regulators for epithelial–mesenchymal transition (EMT) pathway. Similarly, other studies also reported that MTHFD2 could modulate cancer cell migration and invasion through regulating EMT-related proteins [27, 28].

Besides, metabolic alterations in rapidly proliferating cancerous cells might contribute an important role in inducing different phenotypic states. A seminal study on metabolomics analysis showed that somatic cells convert from an oxidative to a glycolytic state in pluripotency, a hall mark of stemness [29]. This stimulated glycolytic pathway foster reprogramming efficiency [30]. Even according to Hanssen et al., the upregulation of glycolysis prior to reactivation of pluripotent markers during metabolic reprogramming [31]. Hence, it is feasible that reprogramming factors first initiate a metabolic shift required to stimulate ancillary endogenic pluripotency factors to complete reprogramming into a stemness state of LCa cells [32]. Further, under low oxygen availability, cancer cells are supposed to shunt glycolytic intermediates into amino acid, lipid and nucleotide synthesis for their cellular proliferation [33]. In a similar fashion, an increased pentose phosphate pathway activity in mouse embryonic stem cells (ESCs), revealed that anabolic glycolysis is common metabolic characteristics both in cancer cells as well as ESCs [34]. Moreover, the upregulated levels of MTHFD2 mRNA under low oxygen tension had been reported in breast cancer [35], which corresponds to our data at protein level. These above-mentioned evidences support the hypothesis that LCa stemness is attributed to up-regulated levels of both HIF-1α and MTHFD2 which establish a positive feedforward loop in parental A549, H1299 and H441 sphere-forming cells, thereby promoting metabolic reprograming and tumor growth. Taken together, our results suggest that MTHFD2 associates stemness state to metabolic state of LCa cells.

Further, many previous studies have focused on malignancy therapeutics that identify pathways which seem to contribute to tumorigenesis and metastasis with more desirable effects and less undesirable adverse effects. In this regard, tumor-initiating cells have been identified in many solid organ malignancies, such pancreas, lung, colon and central nervous system, head and neck squamous cell carcinoma [36–39]. Thus, to elucidate the cells capable of initiating LCa *in vivo*, we subcutaneously injected various dilutions of LCa cells lines in NOD/SCID mice. We also determined that whether MTHFD2 knockdown may influence cancer initiating ability. The results revealed that even 5x10^4^ cellular concentration may drive to tumorigenesis, the aggressiveness of which could be lessened by MTHFD2knockdown, and therefore may be used in developing clinical strategies.

Furthermore, anchorage-independent growth is the capacity of transformed cells to grow independently of a solid surface, is a hallmark of cancer. In this concord, our soft agar assay demonstrated a significantly inhibited anchorage-independent cell growth in MTHFD2-knockdown LCa cell lines, indicating oncosuppressive effect of MTHFD2 knockdown. Further, to examine the *in vivo* effect of MTHFD2 on tumor growth, we injected MTHFD2-knockdown cell lines in mice, which revealed significantly decreased tumor size compared to its parental counterpart.

**Figure 7:**
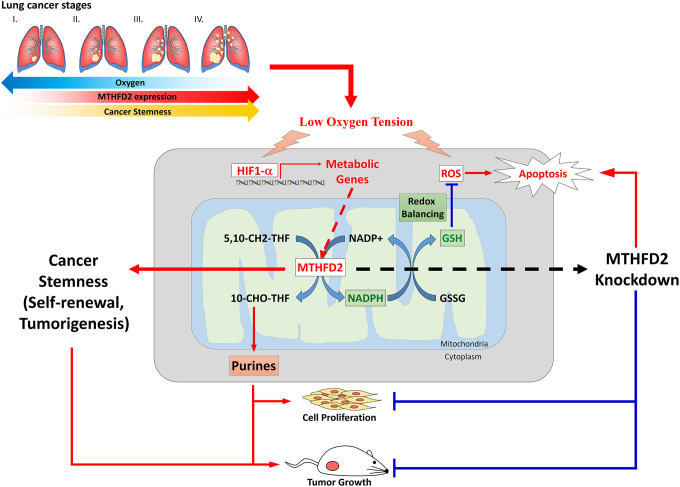
**A schematic pathway of MTHFD2-mediated reprogramming leading to inhibited lung cancer and tumor aggressiveness.**

It has been further reported that mammalian cells under acute exposure of hypoxic conditions (1% O_2_) leads to increased ROS generation at ETC complex III [40]. This imply that low oxygen availability can increase mitochondrial ROS generation, indicating most efficient ETC functions under normoxic conditions. In rapidly growing cancer cells, the surrounding vasculature becomes inadequate with depleted oxygen levels in tissues; as a results, cytochrome oxidase consumes nearly all available oxygen rendering non-mitochondrial compartments essentially anoxic leading to decreased hydroxylase activity [41], and induces stabilization and transcriptional activation of HIF-1α protein, the regulatory member of HIF-1 complex. This HIF then promotes the synthesis of cellular vesicles which facilitates intercellular communication in form of angiogenesis. These evidences infer that decreased cellular oxygen sensing potential in presence of cobalt chloride led to elevated levels of MTHFD2 and highly formed tumors in vector group. It has been suggested that supply of NADPH for anabolic processes might be the rate-limiting step for proliferation of cancerous cells [14, 42, 43]. This was also validated in our results demonstrating suppressed levels of NADPH in MTHFD2-knockdown cancer cell lines. This reduced NADPH supply further led to oxidative stress management in these cancer cells, in terms of increased ROS and cellular apoptosis. Taken together, our data demonstrated that MTHFD2 is significantly associated with LCa and mediates in cancer growth and proliferation, stemness, cellular metabolic reprogramming-dependent tumor aggressiveness, oxygen sensing and oxidative stress response. Eventually, we revealed that MTHFD2 knockdown displays therapeutic activity against LCa (Figure 7), and warrants further clinical investigation.

## MATERIALS AND METHODS

### Cell culture

LCa cell lines A549, H1299 and H441 used in these experiments were purchased from the Bioresource Collection and Research Center, Hsinchu, Taiwan. Cell lines were cultured in RPMI-1640 (Gibco, Grand Island, New York) with 10% (vol/vol) and supplemented with fetal bovine serum (FBS) (Gibco, Mexico) and 1% antibiotic antimycotic solution (Corning, USA). An additional 2.5 μg/ml of puromycin (Sigma-Aldrich, Saint Louis, MO, USA) was supplied in the medium for lentiviral transduced cell lines. The culture medium was maintained at 37°C with 5% CO_2_.

### *In silico* analysis of MTHFD2 gene expression

The Oncomine^TM^ Cancer Microarray Database (https://www.oncomine.org/) was used to perform comparative *in silico* analysis of MTHFD2 gene expression in cancer versus normal tissue. MTHFD2 mRNA expression was queried in cancer vs. normal in the lung cancer category. The query threshold was set at a *p-value* <10^3^, over two-fold difference of expression, top 10% ranking in over-expression genes, and sample sizes are greater than 10. The expression profile of MTHFD2 was further validated in The Cancer Genome Atlas (TCGA) and Genotype-Tissue Expression (GTEx) projects using GEPIA2 online platform (http://gepia2.cancer-pku.cn/#index). We chose dataset of lung squamous cell carcinoma (LUSC), which has been represented as box plot, and *p-value* cutoff is set at 0.05.

### Isolation of total RNA and quantitative real-time polymerase chain reaction

The mRNA levels of MTHFD2 between human normal lung and lung cancer tissues were investigated using Lung Cancer cDNA Array V (OriGene, HLRT105), which consisted of 7-normal, 6-IA, 5-IB, 13-IIB, 7-IIIA, 7-IIIB, and 3-IV with a total of 41cancer tissue samples. The sample patient population includes 29 male and 19 females with the age ranging from 44 to 84 years old. For cellular total RNA was extracted with PureLink® RNA Mini Kit (Thermo Fisher Scientific) and was reverse-transcripted (Applied Biosystems) into cDNA using RevertAid H Minus First Strand cDNA Synthesis Kit (Thermo Scientific). Quantitative PCR was conducted by using 7300 ABI Real-time PCR system (Applied Biosystems) with SYBR Green Master Mix (Applied Biosystems) by using following primers: β-actin-F: 5’-AGAGCTACGAGCTGCCTGAC-3’; β-actin-R: 5’-AGCACTGTGTTGGCGTACAG-3’; MTHFD2-F: 5’-GATGGCCTCCTTGTTCAGTTG-3’; MTHFD2-R: 5’-ATCCTTGTCTGGAGAAACAGCATT-3’

### Gene knockdown by lentiviral transduction

Lentiviral solution was produced by transfecting plasmids pHP, pHEF-VSVG, pGIPZ-shMTHFD2 and pCEP4-tat in 293FT packaging cells (Invitrogen) by using opti-MEM (Gibco) and lipofectamine 2000 (Invitrogen). Infectious viral solution was collected 48h after transfection. Parental cell lines A549, H1299 and H441 were cultured with viral solution with appropriate titers containing 8 μg/ml of polybrene in medium then incubated overnight at 37 °C in 5% CO_2_. For selection of lentivirus transduced cells, cells were cultured in medium with additional 2.5 μg/ml puromycin. The efficacy of lentivirus transduction was evaluated by observing GFP expression under fluorescence microscope. Real-time PCR and western blot analysis were utilized to evaluate the level of MTHFD2 expression. The sequences for MTHFD2 shRNA are as following: shMTHFD2#66: 5’-ATTCCCACATCAATGACTG-3’; shMTHFD2#68: 5’-ATTGCATTTCTATTGGCCT-3’.

### Cell viability and proliferation

Cell viability and proliferation were evaluated by MTT assay. Thousand cells in 100 μl of medium were seeded per well into 96-wells plate and then incubated for 1, 3, 5 and 7 days, and was replaced with fresh medium after every two days. After incubation, medium was discarded and supplied with 100 μl of MTT solution (Sigma, Saint Louis, MO, USA), then incubated at 37°C for 4 hours. After 4-hours incubation, MTT solution was removed and 50 μl of DMSO was added to dissolve the dark blue formazan crystal, and absorbance was determined using ELISA reader (Multiskan RC Microplate Reader, Thermo) at 595 nm.

### Anchorage-independent growth

1 mL of 0.5% agar in complete growth medium was added in each well of six-well plate as a base agar. Top agar was prepared by 1 mL of 0.3% agar in complete growth medium containing 1×10^4^ cells of vector control and MTHFD2-knockdown of A549 and H1299, and 3×10^5^ cells of vector control and MTHFD2-knockdown of H441, top agar was overlaid on the base agar. Growth medium (2 mL) was added on top of the second layer and changed twice a week. After incubation for three weeks, the colonies formed were stained with 0.005% crystal violet in methanol (Fisher Scientific, Hampton, NH, USA), and then enumerated.

### Analysis of cell apoptosis

Flow cytometry-based determination of apoptosis was done by following the manufacturers' instructions of commercial Annexin V-PE / 7-AAD Apoptosis Detection Kit (Elabscience, USA) and the representative histogram were obtained through SA3800 spectral cell analyzer (SONY biotechnology, San Jose, CA, USA).

### Hypoxia induction

Hypoxia was chemically induced by cobalt chloride (CoCl_2_) as described in the previous study [44]. 100 μM of cobalt (II) chloride hexahydrate (Sigma, Saint Louis, MO, USA) was used to treat the target cell lines for 4, 8, 12, 24 and 48 hours. Digoxin (Sigma-Aldrich, D6003) was used as an inhibitor of hypoxia-inducible factor 1-alpha [45]. 100 nM of digoxin was applied to cells with 100 μM of CoCl_2_ for 24 hours. For sphere formation assay under hypoxic condition, cell lines were pre-treated with 100 μM of CoCl_2_ for 48 hours before seeding for sphere formation.

### Sphere formation assay

The medium for sphere formation was DMEM/F12 contained with 1ml of B27 Supplement (50X) (Gibco, NY, USA), EGF 40 ng/ml (Peprotech, USA), bFGF 20ng/ml (Peprotech, USA), Leukemia Inhibitory Factor 20 ng/ml (Sigma, Saint Louis, MO, USA), 100 μl of ITS Supplement (100X) (Sigma, Saint Louis, MO, USA) and 1 % of PSA. For A549, 1 % of extra methylcellulose was added into medium. All the cells were seeded into Ultra-Low Attachment Surface 6 Well Plate (Corning, Kennebunk, ME, USA). An additional 1 ml of medium to the wells was added every 2 days. Cells were incubated at 37°C, 5 % CO_2_ incubator for 1-3 weeks until most of the formed spheres had grown to an adequate size (≥ 100 μm).

### Western blot

Protein extraction and immunoblotting were performed as previously described [46]. Primary antibodies against β-actin (Genetex, #GTX109639, 1:10000), HIF-1α (Genetex, #GTX127309, 1:600), MTHFD2 (Genetex, #GTX115482, 1:1000) were used. Horseradish peroxidase-conjugated secondary antibodies specific to rabbit IgG were used (Genetex, # GTX213110-01, 1:5000). Lastly, membranes were rinsed with Immobilon Western Chemiluminescent HRP Substrate (Merck Millipore, USA) for chemiluminescent detection, then blots were visualized using MultiGel-21 Western/Gel Image System (Top Bio, Taiwan).

### Cellular NADPH determination

To determine cellular NADPH, NADP/NADPH Quantitation Colorimetric Kit (Biovision, #K347) was employed as prescribed by manufacturer’s protocol. For each cell lines, 4×10^6^ cells were seeded and treated with or without 200 μM of CoCl_2_ for 24 hours before cellular NADPH determination.

### Cellular ROS determination

To determine cellular ROS, In Vitro ROS/RNS Assay Kit (Cell Biolabs, San Diego, CA, USA) was used according to manufacturer’s protocol. For each cell lines, 5×10^6^ cells were seeded and treated with or without 200 μM of CoCl_2_ for 24 hours before cellular ROS determination.

### In vivo limiting dilution transplantation assay

All the animal studies were approved by Institutional Animal Care and Use Committee (IACUC) of Taipei Medical University Taiwan (Approval No. LAC-2016-0526). In our study, 6-weeks old non-obese diabetic/severe combined immunodeficiency (NOD/SCID) mice were used for *in vivo* limiting dilution transplantation assay. Mice were anesthetized by mixture of 10X dilution of Zoletil 50 (Virbac, Carros, France) and 10X dilution of Rompun (Bayer, Korea). Both vector control groups and MTHFD2-knockdown groups of A549, H1299 and H441 cells were subcutaneously injected with three different dilutions of cell concentration into the flank of right leg (n=5). The injected cell number of each line were 5×10^5^, 1×10^5^ and 5×10^4^. Tumor size was measured with digital caliper twice a week, and was calculated using following formula:

$$Volume=\frac{Lengh\times Width^{2}}{2}$$

### Statistical analysis

The sample size in each experiment was at least in triplicate, unless otherwise indicated. Statistical analyses were conducted using GraphPad Prism 7 (version 7.00, GraphPad Software, San Diego, CA, USA), and Microsoft Excel (Office 2016 Professional Plus, Santa Rosa, California, USA). All data are presented as mean±SD. A *p-value* of less than 0.05 was considered statistically significant.

## Supplementary Material

Supplementary Figures
