# Supplementary material for: Modulating redox homeostasis and cellular reprogramming through inhibited methylenetetrahydrofolate dehydrogenase 2 enzymatic activities in lung cancer
Source: Aging (Albany NY). 2020 Aug 6;12(18):17930–47. doi: 10.18632/aging.103471 (PMC7585109; doi:10.18632/aging.103471)
Supplement: Supplementary Figures [file aging-12-103471-s001..pdf]

SUPPLEMENTARY FIGURES

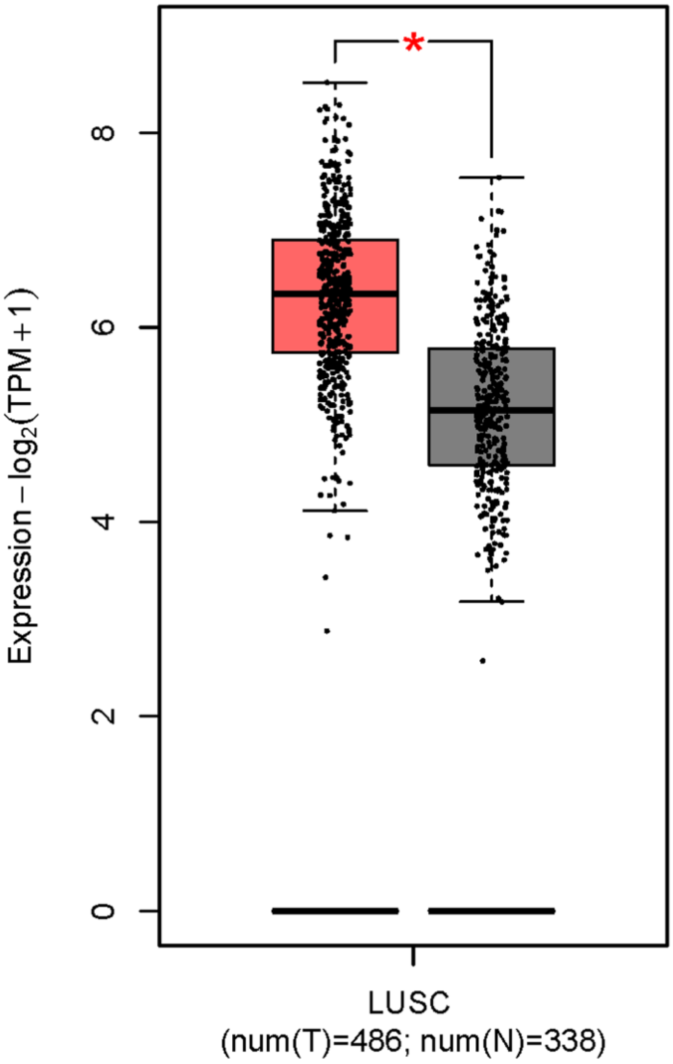

Supplementary Figure 1. *In silico* analysis of MTHFD2 expression profiles from The Cancer Genome Atlas (TCGA) and genotype-tissue expression (GTEx) projects using GEPIA2 online platform (http://gepia2.cancer-pku.cn/#index). The MTHFD2 expression of lung squamous cell carcinoma (LUSC) and its normal counterpart are represented as box plot, and *p-value* cutoff is set at 0.05.

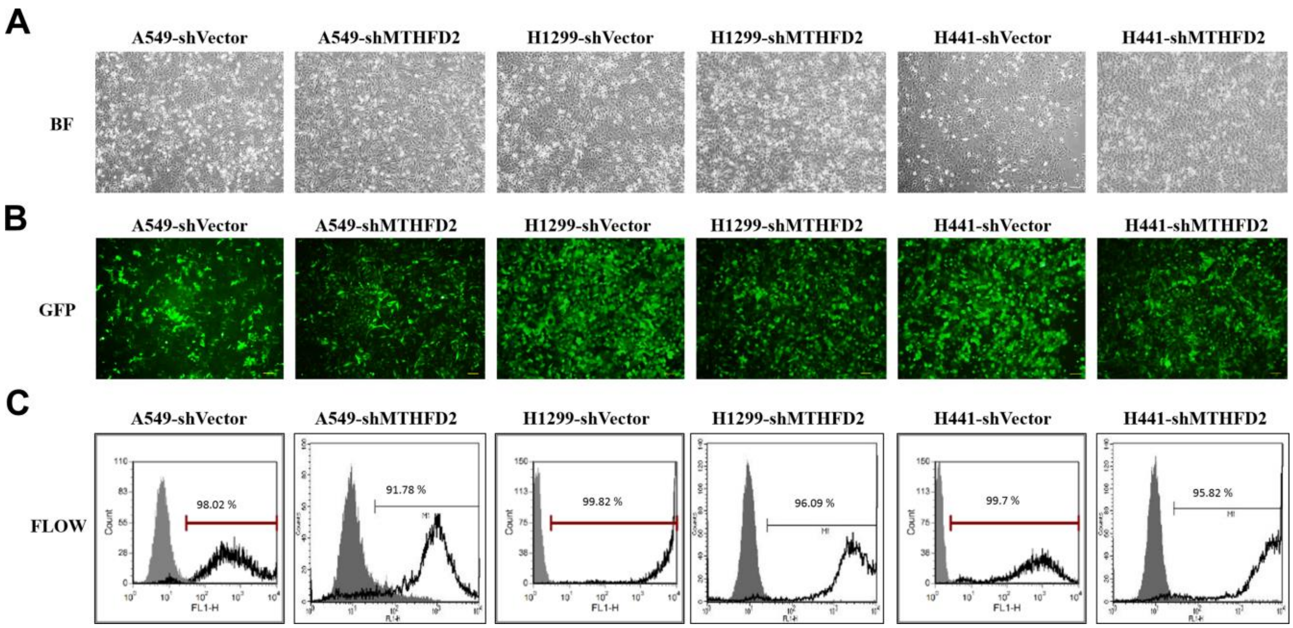

Supplementary Figure 2. Lentivirus transduction in lung cancer cell lines. (**A**) Cell morphology was observed by optical microscope (BF: bright field), and transduction efficiency was analyzed through (**B**) florescence microscopy and (**C**) Flow cytometric analysis in various lung cancer cell lines.

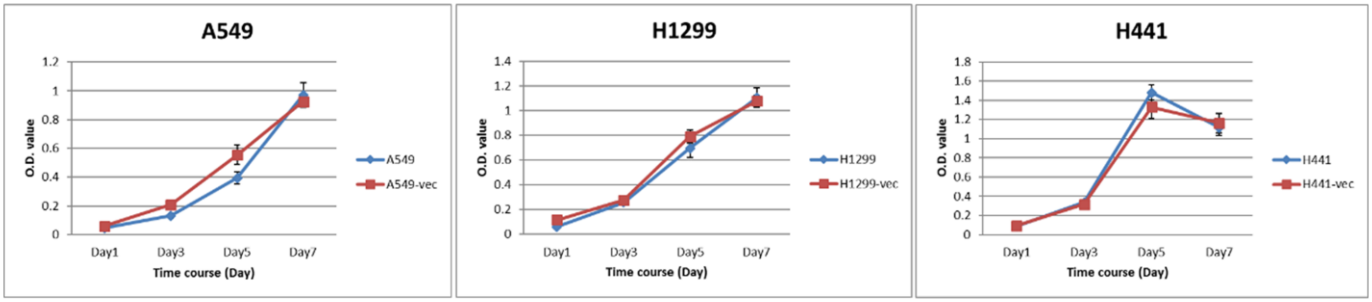

Supplementary Figure 3. MTT assay-dependent cell viabilities of parental and vector control groups of A549, H1299 and H441 cell lines.

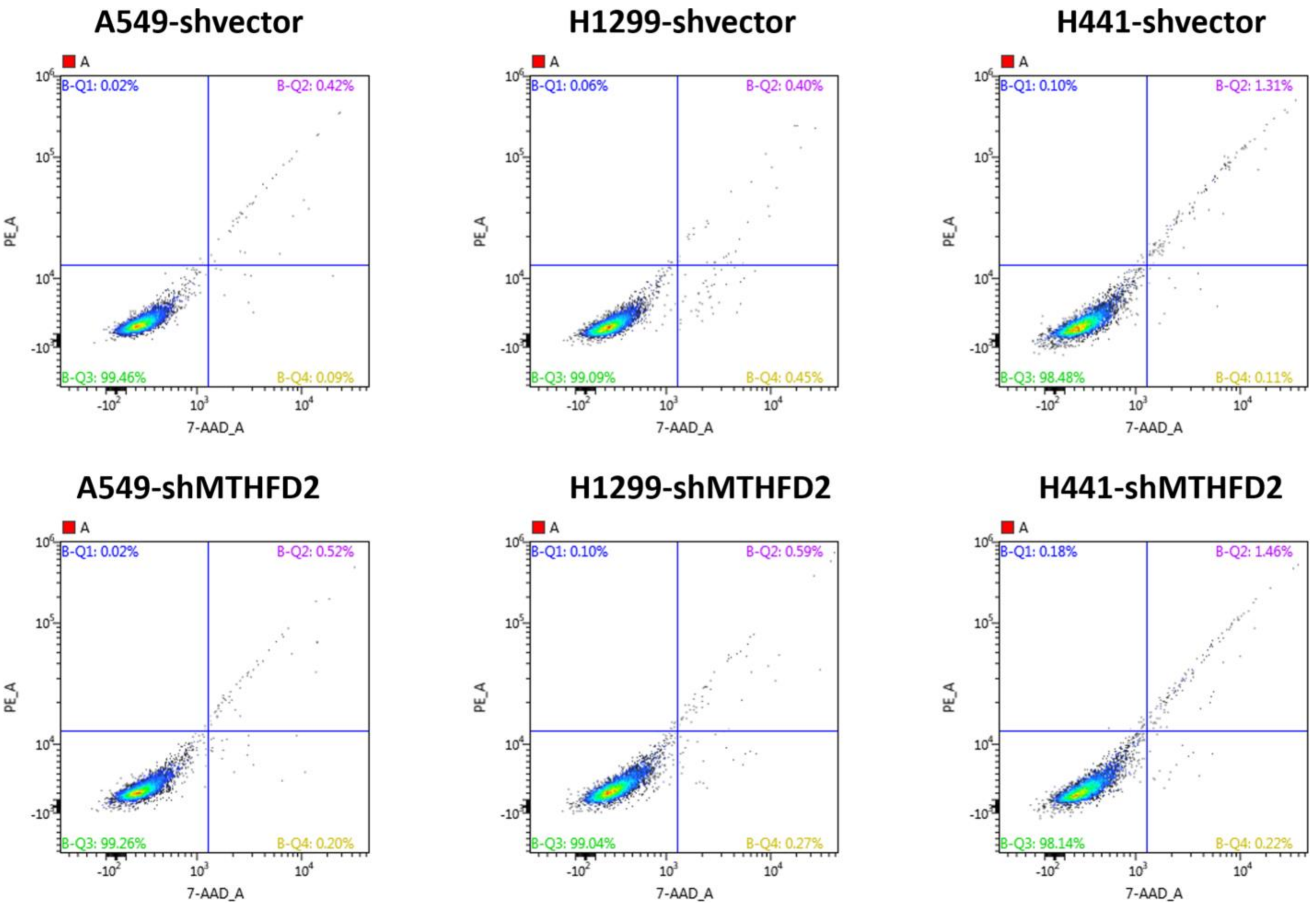

Supplementary Figure 4. Representative results of flow cytometry-based apoptosis assay through Annexin V-PE/7-AAD staining. Data represent the vector control (upper panel) and MTHFD2-knockdown groups of A549, H1299 and H441 (lower panel).

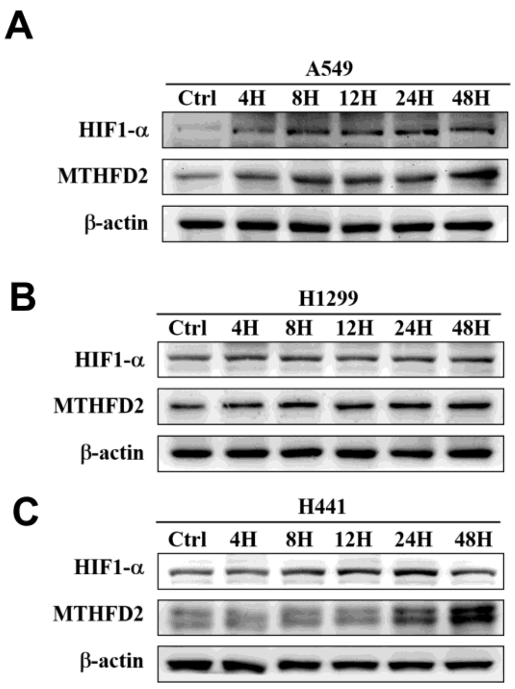

Supplementary Figure 5. Effect of $CoCl_2$ (100 μM)-induced low-oxygen tension on HIF-1α and MTHFD2 protein expression in parental lung cancer cell lines (**A**) A549, (**B**) H1299 and (**C**) H441, which were treated for 0, 4, 8, 12, 24 or 48 hrs. $CoCl_2$: cobalt chloride; HIF-1α: hypoxia inducible factor-1α; MTHFD2: methylenetetrahydrofolate dehydrogenase 2.

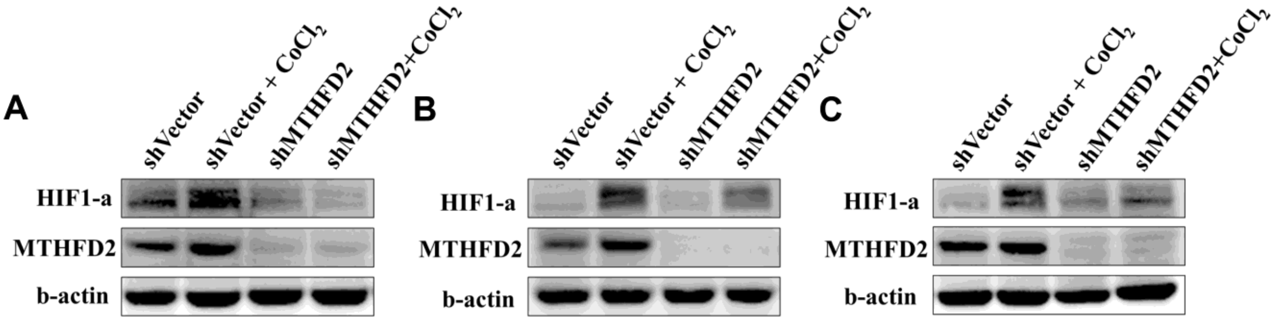

Supplementary Figure 6. Effect of $CoCl_2$-induced low-oxygen tension on HIF-1α and MTHFD2 protein expression in vector control and MTHFD2 knockdown lung cancer cell lines. The vector control and MTHFD2 knockdown of (**A**) A549, (**B**) H1299 and (**C**) H441 cells were treated with 100 μM $CoCl_2$ for 24 h. $CoCl_2$: cobalt chloride; HIF-1α: hypoxia inducible factor-1α; MTHFD2: methylenetetrahydrofolate dehydrogenase 2.
